# Role of RB1 in neurodegenerative diseases: inhibition of post-mitotic neuronal apoptosis via Kmt5b

**DOI:** 10.1038/s41420-024-01955-y

**Published:** 2024-04-18

**Authors:** Shuang Zhao, Guiling Mo, Qiang Wang, Jin Xu, Shihui Yu, Zhibin Huang, Wei Liu, Wenqing Zhang

**Affiliations:** 1 The Innovation Centre of Ministry of Education for Development and Diseases, School of Medicine, South China University of Technology, Guangzhou, 510006 China; 2 Guangzhou KingMed Diagnostics Group Co., Ltd., International Biotech Island, Guangzhou, 510005 China; 3 Greater Bay Biomedical Innocenter, Shenzhen Bay Laboratory, Shenzhen, 518055 China

**Keywords:** Cell death in the nervous system, Apoptosis

## Abstract

During the development of the vertebrate nervous system, 50% of the nerve cells undergo apoptosis shortly after formation. This process is important for sculpting tissue during morphogenesis and removing transiently functional cells that are no longer needed, ensuring the appropriate number of neurons in each region. Dysregulation of neuronal apoptosis can lead to neurodegenerative diseases. However, the molecular events involved in activating and regulating the neuronal apoptosis program are not fully understood. In this study, we identified several *RB1* mutations in patients with neurodegenerative diseases. Then, we used a zebrafish model to investigate the role of Rb1 in neuronal apoptosis. We showed that Rb1-deficient mutants exhibit a significant hindbrain neuronal apoptosis, resulting in increased microglia infiltration. We further revealed that the apoptotic neurons in Rb1-deficient zebrafish were post-mitotic neurons, and Rb1 inhibits the apoptosis of these neurons by regulating *bcl2/caspase* through binding to Kmt5b. Moreover, using this zebrafish mutant, we verified the pathogenicity of the R621S and L819V mutations of human *RB1* in neuronal apoptosis. Collectively, our data indicate that the Rb1-Kmt5b-caspase/bcl2 axis is crucial for protecting post-mitotic neurons from apoptosis and provides an explanation for the pathogenesis of clinically relevant mutations.

## Introduction

The neuroectoderm of the neural plate gives rise to the neuroepithelial cells (NECs, also called neural stem cells) of the neural tube, thus forming the neural precursors that will differentiate into various neurons and glia that comprise the central nervous system (CNS) [1, 2]. Neural stem/precursor cells (NSPCs) are located in the ventricular zone closest to the lumen and can rapidly proliferate to generate excess neural cells during early embryo neurogenesis [3]. Subsequently, approximately 50% of neural cells are cleared by apoptosis before the nervous system matures [4]. This apoptosis process is important for maintaining normal tissue-size homeostasis, removing transiently functional cells that are no longer needed, and ensuring that each brain area has an appropriate number of neurons and glial cells [5, 6]. However, once neurons become mature, their apoptotic capacity is restricted, allowing them to persist in a healthy and functional state throughout life [7]. In certain pathological contexts, the apoptotic pathway of mature neurons can be reactivated [7].

Neurodegenerative disorders affect millions of individuals leading to disability and death. Although neurodegenerative disorders, including Parkinson’s disease (PD), Alzheimer’s disease (AD), Huntington’s disease (HD), and amyotrophic lateral sclerosis (ALS), differ in their pathological genetic changes and degeneration of distinct neuron subsets, the evidence for the activation of apoptotic pathway (such as Caspase-3/8/9, Bax overexpression, and Bcl-2 reduction) in these disorders has been provided by many studies [8–10]. In addition, increasing numbers of studies have provided evidence that there were cell cycle-related proteins that increase the risk of neuron death in AD [11], PD [12], and ALS [13, 14], which suggests the neuronal cell cycle reentry is involved in neuronal apoptosis and death. Interestingly, hyperphosphorylation of RB1, a negative regulator of the cell cycle, is associated with neurodegenerative diseases [15]. Investigators have detected hyperphosphorylation of RB1 in the pathological tissues of AD patients, ALS patients, and PD patients [14, 16–18]. Knockout of *Nrmt1* in mice led to the inactivation of RB1 and eventually induced neurodegenerative diseases [19], while the AD-related gene *presenilin 1* (*PS1*) could protect the anaphase neuronal death by inhibiting the phosphorylation of RB1 [20]. These studies suggested that RB1 may be involved in neuronal apoptosis, but there is no direct in vivo evidence to prove a role for RB1 in the process, and the mechanism underlying the apoptosis pathway remains to be uncovered.

In this study, we recognized *RB1* mutations in 2–3% of neurodegenerative diseases by analyzing clinical sequencing data. By using a Rb1 loss-of-function zebrafish mutant (z*rb1*-KO), we found that Rb1 deletion induced premature apoptosis of post-mitotic neurons through the Kmt5b-Bcl2a/caspase axis. Moreover, we verified the pathogenicity of the R621S and L819V mutations of human *RB1* in neuronal apoptosis. This study clarified the specific effects and molecular mechanisms of Rb1 on activation and regulation of the neuronal apoptosis program and provided a reference for the pathogenesis of Rb1-related neurological diseases.

## Results

### *RB1* mutations existed in several neurodegenerative diseases

**Fig. 1:**
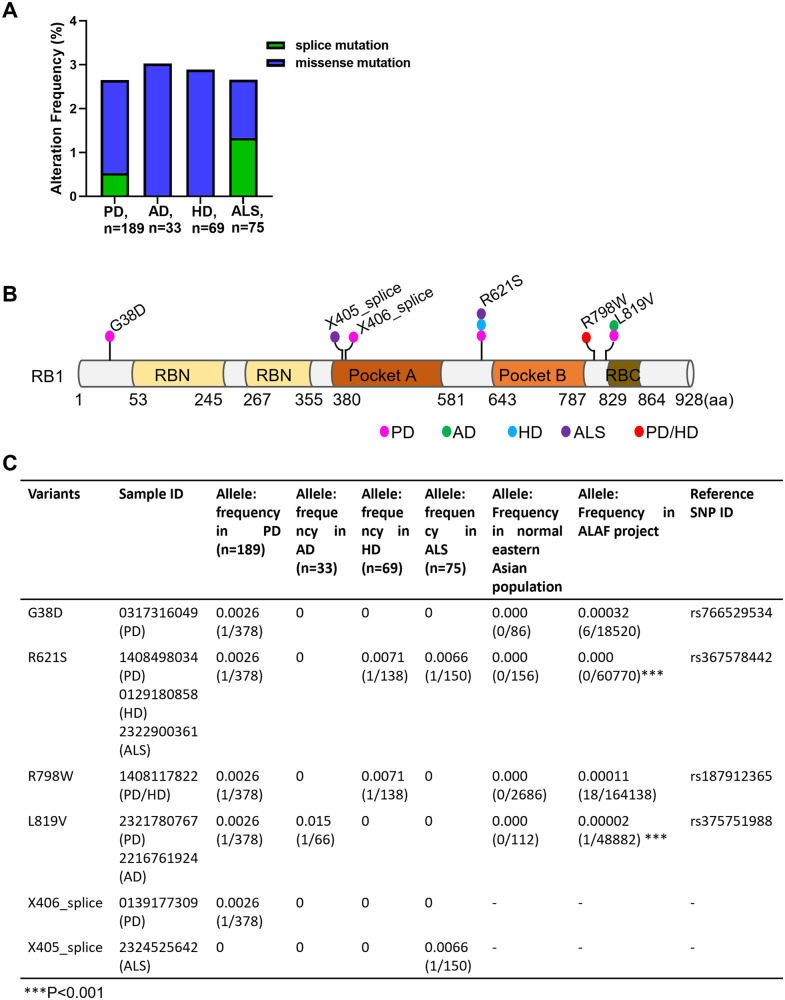
*RB1* was altered in patients with neurodegenerative disease. **A** *RB1* mutation rates by DNA sequence data in blood samples from PD (n = 189), AD (n = 33), HD (n = 69), and ALS (n = 75) patients from the Guangzhou KingMed Diagnostics Group Co. **B** Detailed information of the six mutation sites in neurodegenerative patients. **C** The detailed frequencies of the six mutation sites in neurodegenerative patients, normal Eastern Asian population, and frequency in the ALAF project from NCBI. PD Parkinson’s disease, AD Alzheimer’s disease, HD Huntington’s disease, ALS amyotrophic lateral sclerosis; -: no data. (Fisher’s exact test; ***P < 0.001).

Several studies have reported hyperphosphorylation of RB1 in the pathological tissues of neurodegenerative patients, such as AD, ALS, and PD [14, 16–18]. To determine the relationship between *RB1* mutations and neurodegenerative disease, we calculated the *RB1* mutation rates of PD (189 blood samples), AD (33 blood samples), HD (69 blood samples), and ALS (75 blood samples) using DNA sequence data from Guangzhou KingMed Diagnostics Group Co., Ltd. The results showed that the frequency of *RB1* mutations in the coding region of the protein and RNA splicing was 2.64% in PD, 3.03% in AD, 2.85% in HD, and 2.66% in ALS (Fig. 1A and Table S1). These *RB1* mutations comprise mutations at 6 sites (4 missense mutation sites: G38D, R621S, R798W and L819V; 2 splicing mutation sites: X405 and X406) and were identified in nine neurodegenerative patients (Fig. 1B, C). The frequencies of all four missense alleles in neurodegenerative patients were slightly higher than those in the ALAF project and normal Eastern Asian population from NCBI, but only the R621S and L819V mutations showed statistical significance (Fig. 1C). Taken together, we recognized *RB1* mutations was 2–3% in neurodegenerative diseases by analyzing clinical sequencing data.

### Rb1 deficiency impairs motor ability in juveniles and cognitive ability in adults

**Fig. 2:**
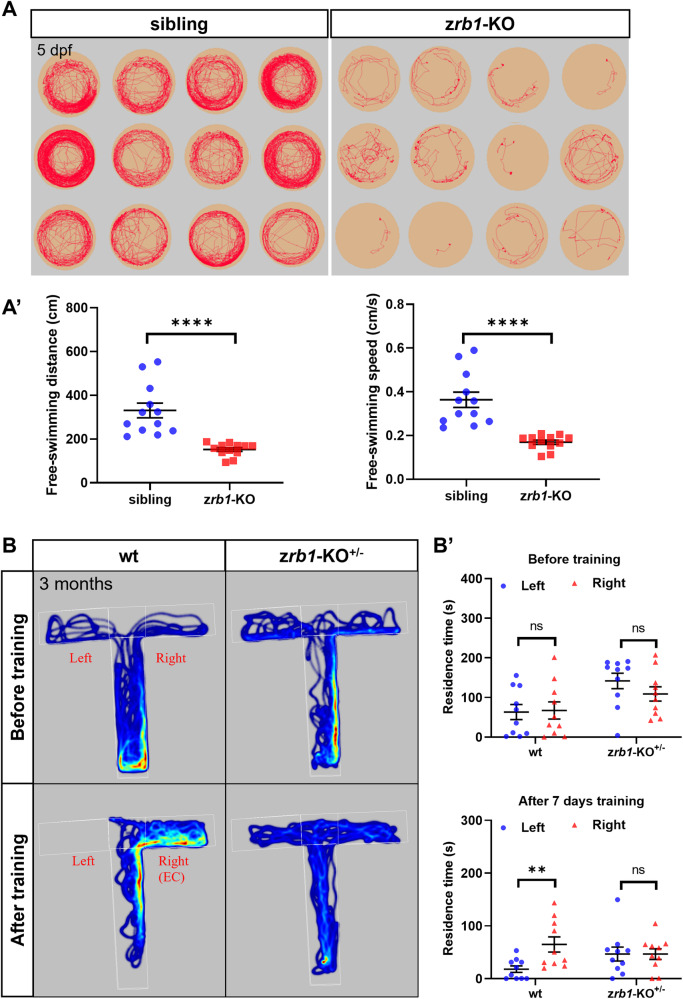
Rb1 deficiency impairs swimming behavior in juveniles and cognitive ability in adults. **A** Tracking from single zebrafish larvae in siblings and z*rb1-*KO mutants at 5 dpf for 15 min. **A**′ The statistical plot of free-swimming distance and free-swimming speed in siblings and z*rb1-*KO mutants at 5 dpf for 15 min (*t*-test; mean ± SEM; ****P < 0.0001; n = 12). **B** The assessment of food stimulus (right arm, EC: enriched chamber) on learning and memory performances of wt and z*rb1*-KO^+/−^ heterozygotes adults in the T-maze test. **B**′. The statistical plot of left and right arm residence time in wt and z*rb1*-KO^+/−^ adult heterozygotes (*t*-test; mean ± SEM; **P < 0.01; ns not significant; n = 10).

Neurodegenerative diseases are characterized by progressive cognitive dysfunction and behavioral abnormalities [21]. To further understand the role of Rb1 in these diseases, we obtain a *rb1* mutant (named z*rb1-*KO) in zebrafish (unpublished results). The z*rb1*-KO mutant had a 2-base deletion in exon 2, which produced a premature stop codon and significantly decreased the expression level of *rb1* RNA (unpublished results). The swimming behavior of z*rb1-*KO homozygous juveniles was studied by the behavioral trajectory tracking system and the cognitive ability of z*rb1-*KO^+/−^ heterozygous adults by T-maze. Our results showed that the swimming distance and speed of z*rb1-*KO homozygous juveniles were significantly reduced (Fig. 2A), indicating that Rb1 deletion caused motor dysfunction in juveniles. Due to the embryonic lethality of z*rb1-*KO homozygous (died at about 15 dpf, unpublished results), we used z*rb1-*KO^+/−^ heterozygous adults (3 months old fish) to test their swimming behavior and cognitive ability. The results showed that the swimming distance and speed of the z*rb1-*KO^+/−^ heterozygous adults were normal (Fig. S1A), but their ability of spatial learning and memory was decreased (Fig. 2B). Before food stimulus training, the wt and z*rb1-*KO^+/−^ heterozygous adults showed no difference in left and right arm residence time. However, after 7 days of food stimulus training in the right arm (enriched chamber: EC), the wt showed increased residence time in the right arm, while the z*rb1-*KO^+/−^ heterozygous adults showed no difference in left and right arm residence time (Fig. 2B). These findings suggest that Rb1 deficiency impairs motor ability in juvenile zebrafish and cognitive ability in heterozygous adult zebrafish.

### Rb1-deficient mutants exhibit a significant hindbrain neuronal apoptosis

**Fig. 3:**
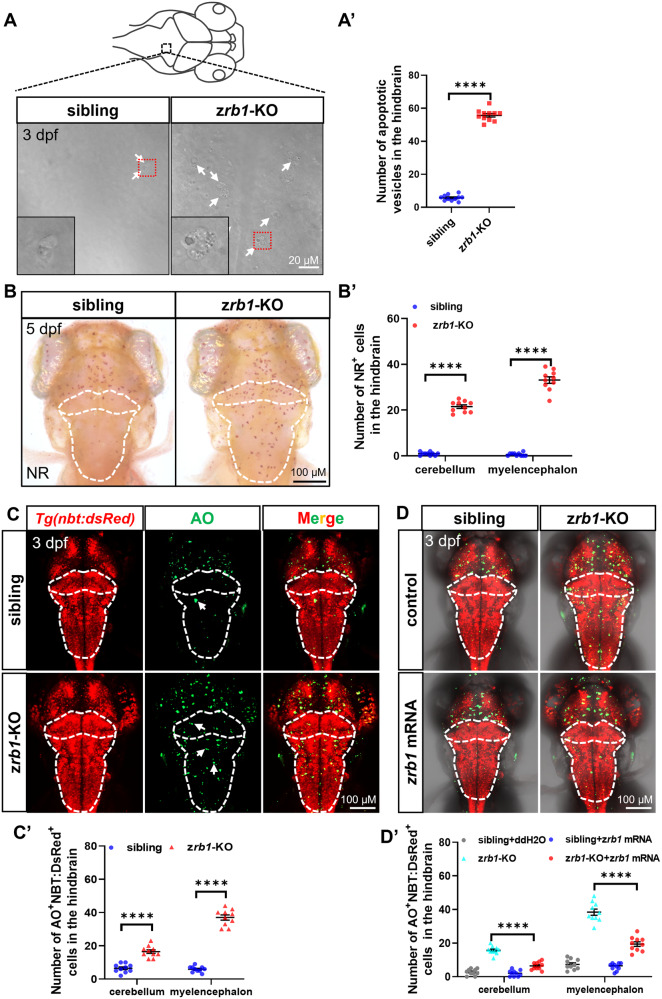
*Rb1* deficiency induced increased neuronal apoptosis of the hindbrain. **A** Apoptotic vesicles in the hindbrain in siblings and z*rb1-*KO mutants at 3 dpf. The white arrows indicate the location of apoptotic vesicles. The image at the bottom left is a magnification of 400 for the red broken line area. **A**′ The statistical plot of apoptotic vesicles in the hindbrain of siblings and z*rb1-*KO mutants (*t*-test; mean ± SEM; ****P < 0.0001; n = 8). **B** Stained by NR to visualize microglia in the cerebellum and myelencephalon of siblings and z*rb1-*KO mutants at 5 dpf. The white dotted line outlines the cerebellum and myelencephalon. **B**′ The statistical plot of NR^+^ cells in the cerebellum and myelencephalon in siblings and z*rb1-*KO mutants (*t*-test; mean ± SEM; ****P < 0.0001; n = 10). **C** Co-staining of AO signals (green) and *Tg(nbt:dsRed)* of siblings and z*rb1-*KO mutants at 3 dpf. The white dotted line outlines the cerebellum and myelencephalon. The white arrows indicate the apoptotic cells. **C**′ The statistical plot of the number of AO^+^/NBT-dsRed^+^ cells in the cerebellum and myelencephalon of siblings and z*rb1-*KO mutants (*t*-test; mean ± SEM; ****P < 0.0001; n = 10). **D** Co-staining with AO (green) and *Tg(nbt:dsRed)* in 3 dpf sibling embryos and z*rb1-*KO mutants after injecting with control and z*rb1* mRNA. **D**′ Quantification of AO^+^/NBT-dsRed^+^ cells of the cerebellum and myelencephalon in all groups of (**D**) (one-way ANOVA; mean ± SEM; **P < 0.01; ****P < 0.0001; n = 10).

As apoptosis is a prominent feature in a broad spectrum of neurodegenerative diseases [22, 23], we investigate the role of Rb1 in neuronal apoptosis. Through the bright-field imaging by confocal, we observed a significant increase in apoptotic vesicles in the hindbrain of the z*rb1*-KO mutants (Fig. 3A). Microglia, the brain’s innate immune cells, can concentrate in areas where neuronal death occurs to eliminate apoptotic cell debris [24, 25]. Therefore, we employed Neutral Red (NR) staining and *apoe* mRNA probe to label microglia and observed an expansion and infiltration of microglia in the cerebellum and myelencephalon of z*rb1*-KO mutants (Fig. 3B and Fig. S2A). Consistent with these findings, AO staining (3 dpf and 5 dpf) and Tunel staining (3 dpf) revealed an increase in neuronal apoptosis in the cerebellum and myelencephalon of the z*rb1*-KO mutants (Fig. 3C, Fig. S2B, C). To further verify whether the induction of hindbrain neuronal apoptosis by *rb1* deletion is an autonomous effect, we injected wild-type z*rb1* mRNA into z*rb1-*KO embryos. The results showed that injection of z*rb1* mRNA in the z*rb1-*KO mutants could partially rescue the neuronal apoptotic and microglia increase phenotype in the cerebellum and myelencephalon (Fig. 3D and Fig. S2D), indicating that the increased apoptosis in the z*rb1-*KO mutants was indeed due to deletion of the *rb1* gene. Taken together, those findings suggest that Rb1-deficient mutants exhibit a significant hindbrain neuronal apoptosis and increased hindbrain infiltration of microglia, and these phenotypes can be partially rescued by wild-type z*rb1* mRNA.

### Apoptotic cells in the z*rb1-*KO are post-mitotic neurons

**Fig. 4:**
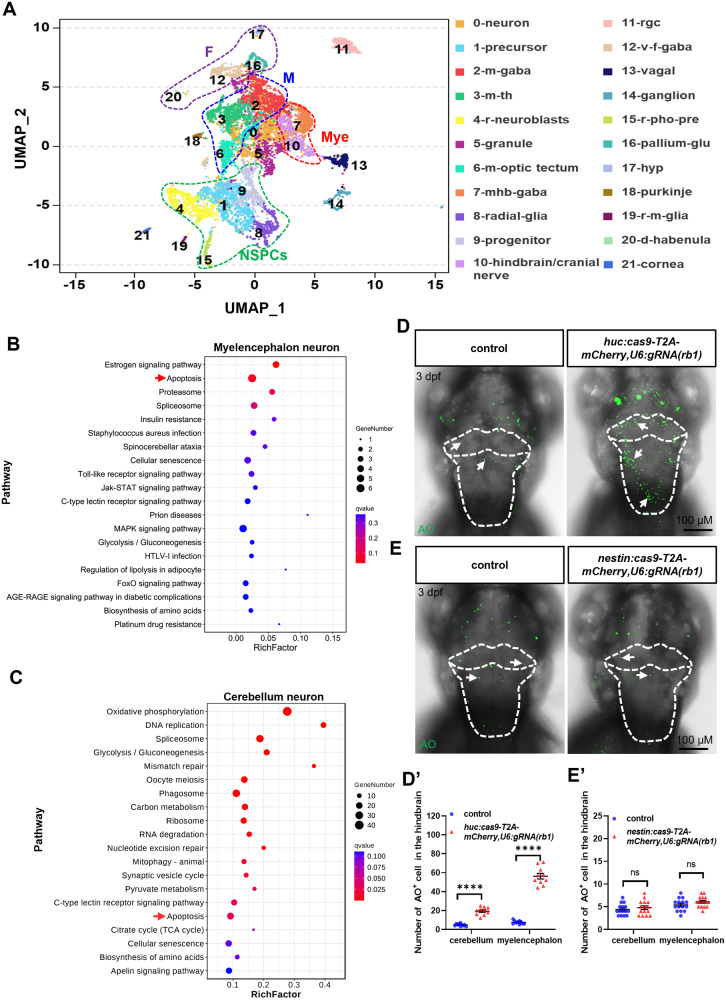
Rb1 regulates the apoptosis of post-mitotic neurons. **A** An UMAP plot re-clustered NSPCs and post mitotic cells into 22 clusters, which were further categorized into 6 subgroups (NSPCs, forebrain neurons, midbrain neurons, cerebellum neurons, myelencephalon neurons, and others) based on their respective locations and differentiation characteristics. NSPCs: 1-precursor, 4-retina neuroblasts (r-neuroblasts), 8-radial glia, 9-progenitors, and 15-retina-photoreceptor precursor cells (r-pho-pre); forebrain (F) neurons: 12-ventral forebrain gabaergic (v-f-gaba), 16-pallium glutamatergic (pallium-glu), 17-hypothalamus (hyp), and 20-dorsal habenula (d-ha); midbrain (M) neurons: 2-midbrain gabaergic (m-gaba), 3-midbrain/thalamus (m-th), and 6 midbrain optic tectum (m-optic tectum); cerebellum (C) neurons: 5-granule and 18-Purkinje; myelencephalon (Mye) neurons: 7-mid-hind boundary-gabaergic (mhb-gaba) and 10-hindbrain/cranial nerves; others: 0-neurons, 11-retinal ganglion cells (rgc), 13-vagal, 14-ganglion, 19-retina-Muller glia (r-m-glia) and 21-cornea. The top 20 functionally enriched KEGG pathways were found in the analysis of DEGs in the myelencephalon (**B**) and cerebellum (**C**). The red arrows indicate apoptosis pathways. Dorsal views of AO staining after wild-type microinjection of *huc:cas9-T2A-mCherry, U6:gRNA(rb1)* (**D**) plasmid and *nestin:cas9-T2A-mCherry, U6:gRNA(rb1)* (**E**) plasmid. The white dotted line outlines the cerebellum and myelencephalon. The white arrows indicate the apoptotic cells. **D′**, **E′** The statistical analysis of AO^+^ cells in the cerebellum and myelencephalon between the control group and microinjection group of (**D**, **E**) (*t*-test; mean ± SEM; ****P < 0.0001; ns, not significant; n ≥ 10).

To characterize the populations of apoptotic neurons in the z*rb1-*KO mutants, we performed 10× single-cell RNA sequencing (scRNA-seq) on whole-brain cells of siblings and z*rb1-*KO mutants at 3 dpf. Based on uniform manifold approximation and projection (UMAP) analysis, we clustered and annotated four major cell types (optic neurons, NSPCs, post-mitotic neurons, and non-neuronal cells), including 28 clusters (Fig. S3A and Table S2). Subsequently, the NSPCs and post-mitotic neuron populations were re-clustered into six subpopulations (NSPCs, forebrain neurons, midbrain neurons, cerebellum neurons, myelencephalon neurons, and others), including 22 clusters (Fig. 4A and Table S3). The Kyoto Encyclopedia of Genes and Genomes (KEGG) pathway-enriched analysis of differentially expressed genes (DEGs) was performed in subpopulations between siblings and z*rb1-*KO mutants. Interestingly, the results showed that the apoptotic pathways were enriched in DEGs of post-mitotic myelencephalon neurons and cerebellum neurons (Fig. 4B and C, Table S4), while cell cycle pathways were enriched in NSPCs populations (Fig. S3B). These data suggest that deletion of Rb1 induces hindbrain post-mitotic neuronal apoptosis as well as NSPCs’ proliferation in the zebrafish.

To further clarify the differences in cell populations in which Rb1 regulates proliferation and apoptosis, we examined the co-localization of proliferating and apoptotic cells in 3 dpf z*rb1-*KO mutants by prolonged BrdU treatment and TUNEL assay. Consistent with the scRNA-seq data, the proliferating and apoptotic cells appeared in different regions (Fig. S3C). Proliferating cells were mainly concentrated in the hindbrain/myelencephalon ventral midline, where neural progenitor cells are located. In contrast, apoptotic cells were scattered in the more mature regions on either side of the midline (Fig. S3C). Furthermore, we modified and obtained two plasmids, *nestin:Cas9-T2A-mCherry,U6:gRNA(rb1)* and *huc:Cas9-T2Am-Cherry,U6:gRNA(rb1)*, which specifically knocked down *rb1* in NSPCs and post-mitotic neurons (Fig. S3D, F), and the expression of *rb1* in the whole brain after injecting two plasmids was significantly decreased (Fig. S3E, G). The results showed that suppressing *rb1* expression in post-mitotic neurons induced only apoptosis in the cerebellum and myelencephalon (Fig. 4D and Fig. S3H), while suppressing *rb1* expression in NSPCs induced cell proliferation but not apoptosis in the cerebellum and myelencephalon (Fig. 4E and Fig. S3I). Taken together, these data suggested that the apoptotic cells in the z*rb1-*KO mutants are hindbrain post-mitotic neurons, and that Rb1 regulates post-mitotic neurons apoptosis independently of its effect on the proliferation of NSPCs.

### Rb1 inhibits Bcl2a/Caspase expression by binding Kmt5b to maintain the survival of post-mitotic neurons

**Fig. 5:**
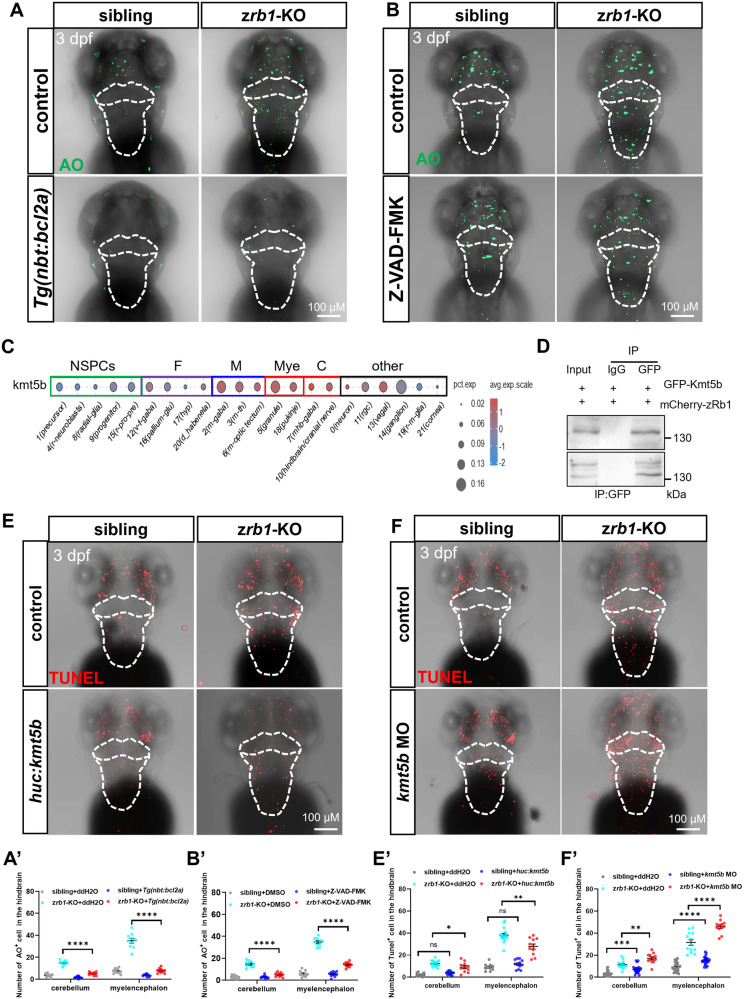
Rb1 regulates post-mitotic neuron apoptosis through the Kmt5b-*bcl2a/caspase* pathway. AO staining in the brain at 3 dpf after overexpression of *bcl2a* in the nervous system (**A**) or treated Z-VAD-FMK (**B**) of siblin*g*s and z*rb1-*KO mutants. The white dotted line outlines the cerebellum and myelencephalon. **A**′, **B**′ Quantification of AO^+^ cells in all groups of (**A**, **B**) (one-way ANOVA; mean ± SEM; ****P < 0.0001; n = 10). **C** Dot plot showing the expression levels of *kmt5b* in six brain regions. The gray level represents the average expression; the dot size represents the percentage of cells expressing the marker genes. **D** Co-immunoprecipitation (Co-IP) analyses on the interaction between zRb1 and Kmt5b. The GPF–Kmt5b and mCherry-zRb1 plasmids were injected into wild-type zebrafish. Immunoprecipitation was performed with an antibody against GPF (upper panel) and confirmed with reciprocal immunoprecipitation experiments with antibodies against mCherry (lower panel). IgG represents a control antibody used for IPs. Input lanes contain lysate equal to one fifth of the amount used for the pull-down assays. IP indicates the antibody used for immunoprecipitation. TUNEL staining in the brain at 3 dpf after injecting *huc:kmt5b-egfp plasmid* (**E**) and *kmt5b* MO (**F**) in siblings and z*rb1-*KO mutants. The white dotted line outlines the cerebellum and myelencephalon. Quantification of TUNEL^+^ cells of the hindbrain after injecting *huc:kmt5b-egfp* plasmid (**E′**) and *kmt5b* MO (**F′**) in siblings and z*rb1-*KO mutants (one-way ANOVA; mean ± SEM; *P < 0.05; **P < 0.01; ****P < 0.0001; n ≥ 10).

Previous studies have shown that E2F family members (mainly E2f1, E2f2, and E2f3) released after Rb1 inactivation could activate ARF, TAp73, and caspase and induce cancer cell apoptosis through p53-dependent or -independent pathways [26–28]. To clarify the apoptotic pathway of Rb1 in the cerebellum and myelencephalon neurons, we crossed z*rb1-*KO mutants with *Tg(nbt:bcl2a)* transgenic fish to overexpress the *bcl2* in the z*rb1-*KO mutants. Meanwhile, we also treated the z*rb1-*KO mutants with the pan-caspase inhibitor Z-VAD-FMK or *p53* MO to inhibit the caspase and P53 pathways. Then, the number of apoptotic cells was detected by AO staining and NR staining. The results showed that either overexpression of *bcl2a* or inhibition of caspase in the z*rb1-*KO mutants decreased the number of apoptotic cells in the cerebellum and myelencephalon (Fig. 5A, B, Fig. S4A, B). However, blocking the P53 pathway did not alter the number of apoptotic cells in the cerebellum and myelencephalon in the z*rb1-*KO mutants (Fig. S4C, D), indicating that Rb1 regulation of neuronal apoptosis depends mainly on the Bcl2 and caspase pathways.

To further clarify the interaction protein of Rb1 involved in neuronal apoptosis, we used MO to knock down *e2f1, e2f2* and *e2f3*, which have been reported as the apoptosis regulator [26, 29]. The MOs for *e2f1* and *e2f3* were designed based on literature reports [30, 31], while the efficacy of the *e2f2* MO was experimentally validated (Fig. S5A, B). The results showed that reducing the expression of *e2f1*, *e2f2*, and *e2f3* does not alleviate the apoptosis in z*rb1*-KO zebrafish (Fig. S5C–E), suggesting that the apoptosis induced by Rb1 appears to be independent of E2f. Then, we used scRNA-seq data to analyze the expression of known binding proteins of Rb1 (e.g., E2Fs, Hdac1, Kmt5b, Dnmt1, etc.). The result showed that only Kmt5b (methyltransferase 5B, Rb1-binding protein that can be enhanced by Rb1 [32]) highly expressed in the post-mitotic neurons of the cerebellum and myelencephalon (Fig. 5C and Fig. S5F). It has been shown that Kmt5b is involved in a neurodevelopmental and intellectual developmental disorder [33]. To investigate whether Kmt5b is involved in the RB1-regulated neuronal apoptosis pathway, we verified the interaction between Rb1 and Kmt5b in zebrafish. We coexpressed full-length mCherry-zRb1 and GFP–Kmt5b in wt zebrafish and performed immunoprecipitation experiment. The result showed that Rb1 can indeed interact with Kmt5b in zebrafish (Fig. 5D). Furthermore, we overexpressed *kmt5b* under the post-mitotic neuron promoter *huc* (*huc:kmt5b-egfp*; Fig. S4G, H) or knocked down *kmt5b* by MO (Fig. S5I) in siblings and z*rb1-*KO mutants. The results showed that overexpression of *kmt5b* in the z*rb1-*KO mutants partially rescued the apoptosis in the cerebellum and myelencephalon (Fig. 5E), while the knockdown of *kmt5b* partially mimicked the phenotype of the z*rb1-*KO mutants (Fig. 5F). Meanwhile, the knockdown of *kmt5b* resulted in decreased expression of *bcl2a* and increased expression of *casp3* and *casp9* in z*rb1-*KO mutants (Fig. S5J). These data indicated that Rb1 could bind Kmt5b to inhibit *bcl2a*/*caspase* expression and regulate the apoptotic pathway to maintain the survival of post-mitotic neurons.

### R621S and L819V mutations of human RB1 may play an important role in neuronal apoptosis

**Fig. 6:**
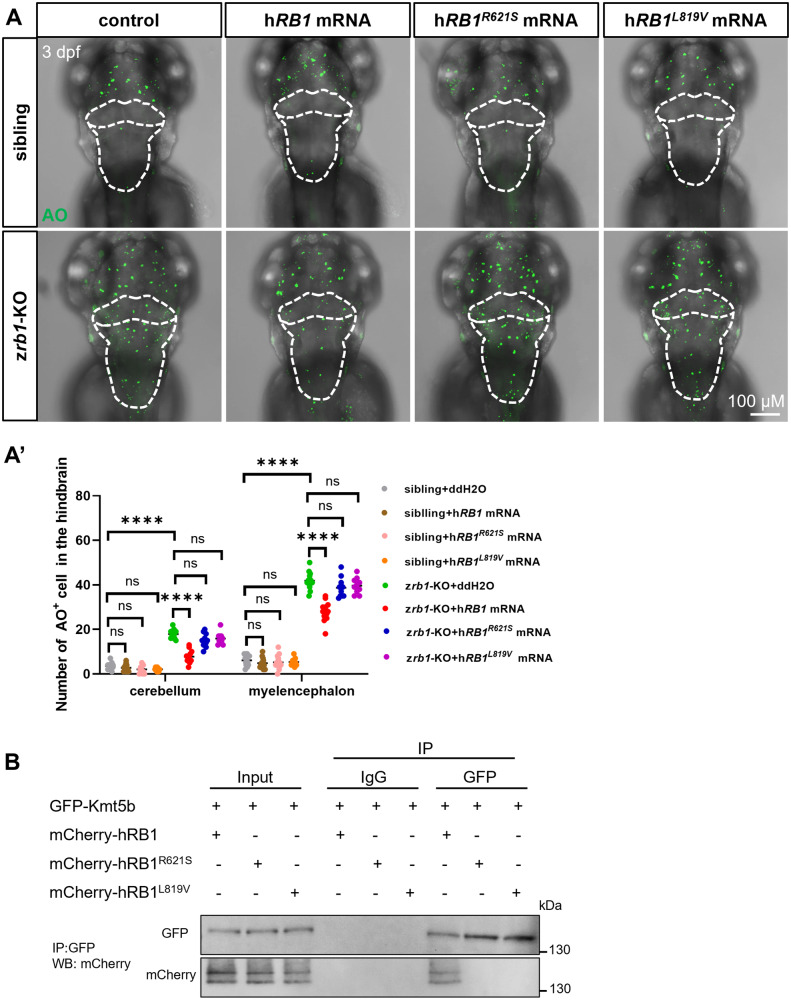
R621S and L819V mutations of RB1 may play a role in neuronal apoptosis. **A** AO staining in the brain at 3 dpf after injecting with control, h*RB1* mRNA, h*RB1*^*R621S*^ mRNA, and h*RB1*^*L819V*^ mRNA in siblin*g*s and z*rb1-*KO mutants. The white dotted line outlines the cerebellum and myelencephalon. **A′** Quantification of AO^+^ cells in all groups of (**A**) (one-way ANOVA; mean ± SEM; ****P < 0.0001; ns, not significant; n ≥ 10). **B** Co-immunoprecipitation (Co-IP) analyses on the interaction between hRB1/ hRB1^R621^/hRB1^L819V^ and Kmt5b. 293T cells were transfected with GPF–Kmt5b and mCherry-hRB1, mCherry-hRB1^R621^ or mCherry hRB1^L819^. Immunoprecipitation was performed with an antibody against GPF (upper panel) and confirmed with reciprocal immunoprecipitation experiments with antibodies against mCherry (lower panel). IgG represents a control antibody used for IPs. Input lanes contain lysate equal to one fifth of the amount used for the pull-down assays. IP indicates the antibody used for immunoprecipitation.

Zebrafish mutants are a powerful tool for the rapid assessment of Variants of Uncertain Significance (VUS) in clinical practice [34]. Neuronal cell death plays a role in many neurodegenerative diseases [22, 23]. Therefore, we used z*rb1-*KO mutants to verify the effect of R621S/L819V alterations (a statistical significance mutation in neurodegenerative diseases, Fig. 1C) in human *RB1* on neuronal apoptosis. The results showed that injecting human *RB1* mRNA into the z*rb1-*KO mutants could partially rescue the neuronal apoptotic phenotype in the cerebellum and myelencephalon (Fig. 6A), while human *RB1*^*R621S*^ mRNA or *RB1*^*L819V*^ mRNA could not alter the number of apoptotic cells in the cerebellum and myelencephalon in the z*rb1-*KO mutants (Fig. 6A). To demonstrate whether the h*RB1*^*R621S*^ and h*RB1*^*L819V*^ mutation abrogates interaction between RB1 and Kmt5b, we perform immunoprecipitation assay in 293T cells. The result showed that hRB1 can interact with Kmt5b, while the h*RB1*^*R621S*^ and h*RB1*^*L819V*^ mutations fail to interact with Kmt5b (Fig. 6B). This suggests that the h*RB1*^*R621S*^ and h*RB1*^*L819V*^ mutations may abolish the interaction between RB1 and Kmt5b, resulting in an ineffective rescue of apoptotic phenotypes in z*rb1*-KO. The above results suggest that R621S and L819V mutations of human *RB1* may play a role in neuronal apoptosis and are associated with neurodegenerative diseases.

## Discussion

To clarify the role of RB1 in neuronal apoptosis and neurodegenerative diseases, we first analyzed blood sequencing data from patients with neurodegenerative diseases. The results revealed *RB1* mutations in 2–3% of these patients. By constructing the zebrafish Rb1-deficient model, we found that there was significant post-mitotic neuronal apoptosis in the hindbrain, which was regulated by Caspase/Bcl2 rather than P53. We further demonstrated that overexpression of Kmt5b in post-mitotic neurons could partially rescue the Rb1 deficiency-induced apoptosis, suggesting a crucial role of the Rb1-Kmt5b-Caspase/Bcl2 axis in regulating post-mitotic neurons.

As the first cloned tumor suppressor gene, numerous studies have documented the anti-proliferative function of Rb1 in the nervous system [35–37]. However, the role of Rb1 in regulating apoptosis in the nervous system is still highly controversial. Neuronal apoptosis in *Rb1*-KO mice has been partly attributed to developmental defects in extra-embryonic and hematopoietic tissues [37, 38]. The researchers then conditionally knocked out *Rb1* in different nerve cells, which resulted in different apoptotic outcomes. For example, deleting *Rb1* in NSPCs (*Nes*^*+/cre*^*Rb*^*lox/lox*^) did not affect cell apoptosis in CNS [37], while deleting *Rb1* in telencephalic neurons (*Foxg1*^*+/cre*^*Rb*^*lox/lox*^) and glial cells (*Gfap*^*+/cre*^*Rb*^*lox/lox*^) could induce different degrees of apoptosis in CNS [35, 39]. Moreover, the neuronal types and mechanisms by which Rb1 induces apoptosis remain unclear. Our findings suggest that Rb1 autonomously protects post-mitotic neurons via the Kmt5b-Bcl2a/Caspase pathway.

Our data suggest that after neurons exit the cell cycle, Rb1 inhibits apoptosis by activating Kmt5b to suppress downstream Caspase activation. It is possible that, under normal circumstances, the Kmt5b separated from Rb1 to activate Caspase expression in the apoptosis of excess or temporarily functional neurons. However, reducing Kmt5b or Rb1 expression can lead to inappropriate apoptosis of post-mitotic neurons. This is similar to our previous finding that Rb1-E2F1 can protect T lymphocytes from premature apoptosis in zebrafish [30]. We speculate that Rb1 may play an essential role in activating and mediating apoptosis in various tissues that require strict control of cell number and quality.

Previous studies have shown that RB1 can recruit and stabilize KMT5B (a type of arginine methyltransferase) to the genome to methylate histone H4 at lysine-20 (K20), thus inhibiting gene transcription [32, 33]. Clinical studies have shown that pathogenic variants of *KMT5B* are associated with global developmental delay, macrocephaly, autism, and congenital abnormalities (OMIM# 617788). In mice, *Kmt5b* knockout is embryonic lethal [40], and monoallelic pathogenic variants of KMT5B disrupt normal epigenetic regulation of neural development [41]. In *Kmt5b*^*+/*−^ and *Kmt5b*^*−/−*^ mice brains, an increased number of cell death was observed in the cortex and corpus callosum [41]. In this study, through analyzing single-cell sequencing data of the whole brain of zebrafish at 3 dpf, we found that *kmt5b* was more highly expressed in neurons differentiated in the midbrain and hindbrain than in NSPCs. Knockdown of *kmt5b* caused apoptosis of hindbrain neurons similar to *zrb1*-KO mutants, while overexpression of *kmt5b* partially rescued the Rb1-deficient phenotype. However, the binding site between Kmt5b and Rb1 and the epigenetic regulation pathway regulated by the Rb1-Kmt5b axis remains to be studied.

Clinical studies have shown that the dysregulation of RB1 and its pathways has been detected in some glioblastoma and neurodegenerative disease samples [15, 18, 42, 43]. Consistent with this, we also detected the rate of *RB1* heterozygous mutations in blood samples from neurodegenerative disease patients. Interestingly, the type of mutations in *RB1* was different in glioma patients and neurodegenerative patients. Deep deletion and truncation were the predominant mutations in glioma patients (data not shown), while missense substitution was predominant in neurodegenerative patients. It is possible that different types of *RB1* mutations can induce proliferative and apoptotic neurological diseases by altering different binding proteins. For example, mutations near the LXCEX binding site sequence (amino acids 709–757) on the RB1 pocket 2 may alter RB1’s binding to KMT5B and induce neuronal apoptosis, while mutations in the E2Fs binding site (amino acids 467–548) may more significantly affect cell proliferation. To confirm this association, stable, heritable, and feasible site-directed mutant zebrafish and mouse strains need to be established for further analysis.

Apoptosis is an important process in the development of the nervous system. Typically, about 50% of neurons die during neurogenesis before the nervous system matures, and this process is critical for establishing a definite pattern of neuronal connections [44]. This is commonly known as neurotrophic cell death which is regulated through the competition of a limited amount of nerve growth factor (NGF) released by the target cells these neurons innervate [45, 46]. Multiple neurotrophins and their receptors that signal to promote survival have now been identified. These include the classical neurotrophins NGF, and their respective ligands Trk family (TrkA, TrkB, and TrkC) and P75 neurotrophic receptor [47–49]. P75 neurotrophic receptor is an important receptor for the role of neurotrophins in modulating brain plasticity and apoptosis [50]. It has been found that the expression pattern of the neurotrophic factor itself has not changed significantly in *Rb1* mutants. In contrast, the expression of the low-affinity NGF receptor p75 and the high-affinity NGF receptor TrkA were significantly reduced [51]. The lack of differentiation and increased neuronal cell death in sensory ganglia in RB1-deficient embryos appear to be at least partially explained by the lack of TrkA and p75 proteins [51]. In addition, after treating human glial cells and mouse cortical neurons with neurotrophic factors, elevated cytoplasmic E2F1 and RB1 nuclear phosphorylation levels were observed [52, 53]. Therefore, RB1 protein and its partner may play an important role in the establishment of a brain neurotrophin-mediated apoptosis signal pathway.

## Materials and methods

### Patients

Patients with neurodegenerative disease between 2017 and 2020 from Guangzhou KingMed Diagnostics were retrospectively retrieved and their test results and histological follow-up results were collected and analyzed. All patients provided written informed consent. Approval was obtained from the ethics committee of Guangzhou KingMed Diagnostics (Reference number: 2022091).

### Mutation analysis

The Single Nucleotide Polymorphism database (dbSNP) (https://www.ncbi.nlm.nih.gov/snp) was referred to for extracting the frequencies of variants in the normal Eastern Asian population, and Allele Frequency Aggregator Project (https://www.ncbi.nlm.nih.gov/snp) was referred to for evaluating the frequencies of variants in the whole population.

### Fish maintenance

Zebrafish (Danio rerio) were maintained at 28.5 °C on a 14 h light/10 h dark cycle. The following strains were used: AB, *Tg(nbt:dsRed)* [24], *Tg(nbt:bcl2a)* (also named *Tg(Xla. Tubb:bcl-2)*) [54], and z*rb1-*KO (F3, loss of function mutant, unpublished results) mutants.

### Cell culture and transfection

293T cells were from Cell Bank/Stem Cell Bank Chinese Academy of Sciences. For 293T cell culture, cells were maintained in Dulbecco’s modified Eagle’s medium (DMEM, Gibco, 11965092) supplemented with 10% fetal bovine serum (FBS, Gibco, 10270106). Cells were plated in 6-well culture plates and at 70–90% confluence they were transfected with GFP-Kmt5b and mCherry-hRB1/mCherry-hRB1^R621S^/mCherry-hRB1^L819V^ for 48 h. Cells were cultured at 37 °C in a 5% CO_2_, humidified atmosphere. Lipofectamine 3000 (Invitrogen, L3000075) was used to transfect the cells.

### Neutral red staining and whole mount in situ hybridization

Microglia cells were scored in live larvae by treatment with vital dye neutral red (NR, Sigma-Aldrich, N6264) and whole mount in situ hybridization as described previously [55–58]. Briefly, embryos were stained with NR in egg water with 2.5 μg/mL neutral red and 0.0045% PTU at 28.5 °C for 4–6 h, followed by 3–4 egg water changes, and then analyzed 3–4 h later using a stereomicroscope (ZEISS, Axio Zoom.V16).

### Acridine orange staining and TUNEL staining

The apoptosis of live zebrafish embryos was determined by acridine orange (AO, Sigma-Aldrich, A6014). To experiment, the embryos were placed in 1 µg/mL of AO egg water at 28.5 °C for 1 h. After washing six times/5 min in egg water, embryos were anesthetized with tricaine, mounted in 1% low melting point agarose, and examined using a Zeiss LSM800 laser scanning confocal microscope. The TUNEL assay was performed using the in situ Cell Death Detection Kit TMR Red (Roche 12156792910) according to the manufacturer’s instructions.

### Co-immunoprecipitation

For zebrafish, about 100–200 2 dpf embryos (inject with GFP-Kmt5b and mCherry-zRb1) were collected and 1 ml deyolk buffer (0.3 mM PMSF, 1 Mm EDTA in 1×PBS) was added for deyolk. Then centrifuge 300 g/4 °C for 5 min to remove the supernatant. Add 90 μl 2× cell lysis buffer (CST, 9803), ice lysis for 10–30 min. Added with equal volume of H_2_O (cocktail and PMSF), centrifuge at 4 °C, 14,000 × *g* for 5 min, and transfer the supernatant to the new EP tube. 293T cells were transfected GFP-Kmt5b and mCherry-hRB1/mCherry-hRB1^R621S^/mCherry-hRB1^L819V^ for 48 h. Transfected cells were lysed in 1x cell lysis buffer (1 mM PMSF/1x cocktail), ice lysis for 10–30 min. Then centrifuge at 4°C, 14,000 × *g* for 10 min, and apply the supernatant to the new EP tube. Zebrafish sample and cell sample supernatants were incubated overnight at 4 °C with 5 μg of anti-GFP antibody (Abcam, ab6658) or control IgG (Proteintech, 30000-0-AP), followed by incubation at 4 °C for 1–2 h with dynabeads protein G (Invitrogen™, 10004D). Beads were washed with cell lysis buffer and resuspended in 1× SDS–PAGE loading buffer for western blot analysis.

### Western blot

Use 6% SDS-PAGE gel, and the separated proteins were transferred by electro blotting to NC membranes. The membranes were blocked with 5% non-fat dry milk in TBST and incubated with the primary antibody including anti-GFP antibody (Abcam, ab6658) and anti-mCherry antibody (Abcam, ab125096) overnight at 4 °C. Then washing three times, the second antibody including anti-Goat HRP (Proteintech, SA00001-3) and anti-mouse HRP (Proteintech, SA00001-8) for about 2 h Immunolabelling was detected using SuperSignal West Femto (Thermo Fisher Scientific, 34096).

### The 10× single-cell sequencing (scRNA-seq) sample preparation, sequencing, and analysis

The whole brains of siblings and z*rb1-*KO mutants (each group n = 10) were isolated under a microscope using the needle of a 1.5 ml syringe at 3 dpf. Brain tissues were dissociated into single cells using a papain solution (Worthington, LS003126) as described previously [59]. After staining the cells with trypan blue, they were counted. The proportion of live cells was calculated to ensure that the proportion of live cells was ≥ 90%. The cell concentration was adjusted to 1000 cells/μL. Single-cell encapsulation, cDNA library synthesis, and RNA sequencing were completed by Gene Denovo Biotechnology Co., Ltd (Guangzhou, China). Raw sequencing data were processed by the Cell Ranger (v7.0) provided by 10X Genomics with default options. Reads were aligned to the zebrafish reference transcriptome (Ensembl release 104). Seurat was then used for further quality control and analysis of the single-cell transcriptome data. The scRNA-seq datasets contained a total of 17356 high-quality cells, including 8557 sibling and 8799 z*rb1-*KO mutant cells with a median of ∼3645 unique molecular identifiers (UMIs) detected per cell and ∼1156 genes detected per cell. After cell clustering and Uniform Manifold Approximation and Projection (UMAP) visualization [60], singleR was used for cell annotation [61]. The markers used for cell clustering have been obtained in previous reported literature [59, 62] and are listed in Table S3. KEGG pathway analysis [63] and other bioinformatics analyses were performed using Omicsmart, a real-time interactive online platform for data analysis (http://www.omicsmart.com).

### Plasmid construction and microinjection

We obtain the *Cas9-T2A-mCherry,U6:gRNA* plasmid from Prof. Yang [42]. The *nestin* and *huc* promoters with Xhol and Agel sites were as previously studied [64, 65], and cloned into the *Cas9-T2A-mCherry,U6:gRNA* vectors using ClonExpress® Ultra One Step Cloning Kit (Vazyme, C115). In addition, the sequences of *kmt5b, huc* promoter, and *egfp* fragment were amplified by PCR and spliced into pTol vector using ClonExpress® ULtra One Step Cloning Kit (Vazyme, C115-01) to construct the *huc:kmt5b-egfp* plasmid. All plasmids were injected into the embryos at the one-cell stage at a concentration of 20 ng/uL. Following microinjection, all embryos were raised in egg water at 28.5 °C.

### Morpholino and RNA injections

The design and injection morpholino of *e2f1* [30], *e2f3* [31], *kmt5b* [66] and *p53* [67] were performed as previously reported. The *e2f2* MO (5′-ATTCAGTCAGGCACACTTACAGCCA-3′) used to block splicing was designed and obtained from Gene Tools. The MO sequences used in this study are listed in Table S5. The *e2f1* (0.5 mmol/L), *e2f2* (0.5 mmol/L), *e2f3* (0.5 mmol/L), *kmt5b* (0.5 mmol/L) and *p53* (0.5 mmol/L) MOs were injected into zebrafish embryos at the one-cell stage. The mRNA synthesis was performed using the mMESSAGE mMACHINE™ SP6 Transcription Kit (Invitrogen, AM1340) according to the manufacturer’s instructions. The z*rb1* mRNA, h*RB1* mRNA, h*RB1*^*R621S*^ mRNA, and h*RB1*^*L819V*^ mRNA were pressure injected into one-cell-stage embryos at a concentration of 125 ng/μL and collected at the appropriate stage.

### Zebrafish behavioral trajectory tracking and T-maze

The embryos and adult fish were placed in 48 well plates at 5 dpf or in the 1 L aquarium at 3 months to detect behavior trajectory by Zebrafish behavioral trajectory tracking system (DanioVision, Noldus) as previously reported [68]. For the T maze as previously described [69]. In brief, the 3 months old adult wt and z*rb1-*KO^+/−^ heterozygotes were used for training for 7 days. During the food stimulus training, the food was placed in the right arm to form an enriched chamber (EC), and the wt and z*rb1-*KO^+/−^ heterozygotes were placed in the T-maze, trained for 5 min, and the residence time of fish to right arm (EC) and left arm was observed and recorded. If the fish does not find the EC zone after 5 min, guide it into the EC zone and stay for 3 min. After 7 days of training, the food was removed from the right arm, and the residence time in the left and right arms was measured.

### Statistical methods

Statistical analysis was performed using GraphPad Prism 9. The unpaired two-tailed Student’s *t*-test is used to compare the means between the two groups. The one-way analysis of variance (ANOVA) test followed by Tukey’s or Bonferroni adjustment is used for multiple comparisons. Fisher’s exact test was used to analyze two categorical variables. In each graph, the error bars reflect the mean ± SEM. Differences with *P* < 0.05 (^*^) or *P* < 0.01 (^**^) or *P* < 0.001 (^***^) or *P* < 0.0001 (^****^) were considered statistically significant.

### Supplementary information

SUPPLEMENTAL MATERIAL

Table S1

Table S2

Table S3

Table S4

Table S5

original western blot

## Data Availability

All data are presented in the main text or Supplementary Materials. Data produced in this manuscript are available on Gene Expression Omnibus (GEO) with accession number GSE212888.
