# Supplementary material for: Role of RB1 in neurodegenerative diseases: inhibition of post-mitotic neuronal apoptosis via Kmt5b
Source: Cell Death Discov. 2024 Apr 18;10:182. doi: 10.1038/s41420-024-01955-y (PMC11026443; doi:10.1038/s41420-024-01955-y)
Supplement: Supplementary file 1 — SUPPLEMENTAL MATERIAL [file 41420_2024_1955_MOESM1_ESM.docx]

**Role of RB1 in neurodegenerative diseases: Inhibition of post-mitotic neuronal apoptosis via Kmt5b**

**Supplementary information**

Shuang Zhao ^1^; Guiling Mo ^2^; Qiang Wang ^1^; Jin Xu ^1^; Shihui Yu ^2^; Zhibin Huang ^1^; Wei Liu ^1^*; Wenqing Zhang ^1,3^*

^1^ The Innovation Centre of Ministry of Education for Development and Diseases, School of Medicine, South China University of Technology, Guangzhou 510006, China

^2^ Guangzhou KingMed Diagnostics Group Co., Ltd., International Biotech Island, Guangzhou, 510005 China

^3^ Greater Bay Biomedical Innocenter, Shenzhen Bay Laboratory, Shenzhen 518055 China

^*^**Corresponding authors:**

W.Q.Z.: The Innovation Centre of Ministry of Education for Development and Diseases, School of Medicine, South China University of Technology, Guangzhou 510006, China. Tel/Fax: 86-20-39380971; Email: mczhangwq@scut.edu.cn.

W.L.: The Innovation Centre of Ministry of Education for Development and Diseases, School of Medicine, South China University of Technology, Guangzhou 510006, China. Tel/Fax: 86-20-39380971; Email: [liuwei7@scut.edu.cn](mailto:liuwei7@scut.edu.cn).

**Materials and Methods**

**BrdU assay and TUNEL assay**

Usually, embryos at the appropriate stage were incubated in 10 mmol/L bromodeoxyuridine (BrdU, Sigma-Aldrich, B9285) for 4 h at 28.5˚C, then incubated in egg water with 0.0045% PTU for about 30 minutes, and subsequently fixed in 4% paraformaldehyde. For BrdU and TUNEL co-staining, embryos were incubated with BrdU for 2 h, changed in egg water with 0.0045% PTU for about 2 h, and then fixed in 4% paraformaldehyde. According to the manufacturer’s instructions, the TUNEL assay was performed using the in situ Cell Death Detection Kit TMR Red (Roche 12156792910). After the TUNEL assay, the embryos were treated with 2 N HCl for about 60 minutes, stained with primary mouse anti-BrdU (Roche, 11170376001; 1:400, at 4°C overnight), and then visualized with Alexa Fluor 488 donkey anti-mouse (Abcam, ab150109) antibodies.

**Mutant identification**

The zebrafish z*rb1*-KO mutants were genotyped by PCR with high-resolution melting (HRM, FP, 5′-GCAATGTCAACCATGTCTGTC-3′; RP, 5′-AAGTGCGGTAAAGCAGATATC-3′) analysis. Briefly, the PCR products were subjected to the first round of HRM analysis. Wild-type (wt) and mutants showed one peak and heterozygotes showed two different peaks. Next, we added pre-prepared wt PCR products to all products for the second round of HRM analysis. The wt PCR products showed one peak and the PCR products of heterozygotes and mutants showed two different peaks. The two results were compared to confirm the mutants.

**Quantitative real-time PCR**

RNA was extracted from the whole body and the whole brain of zebrafish. The RNA was extracted using Trizol (Invitrogen, 15596018) and cDNA was generated by M-MLV reverse transcriptase (Promega, M1701). Quantitative reverse-transcription PCR (qRT-PCR) was performed using the light cycler 96 system (Roche, 05815916001) with a SYBR Green master mix (Roche, 06402712001). The primer sequences are listed in Table S5.

**Figures**

**
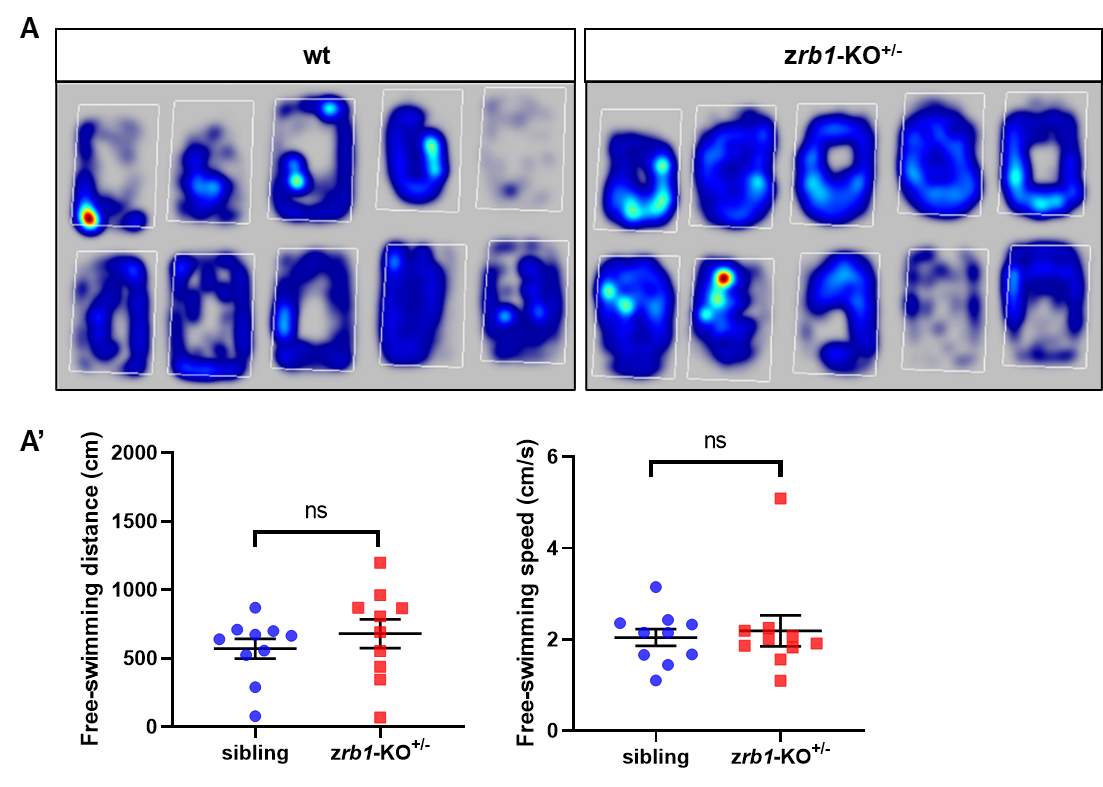
**

**Fig. S1. The z*rb1*-KO^+/-^ heterozygotes adults have normal swimming behavior. (A)** Tracking from single zebrafish in wt and z*rb1*-KO^+/-^ adult heterozygotes (heat map) at 3 mouth for 5 min. **(A’)** The statistical plot of free-swimming distance and free-swimming speed in wt and z*rb1*-KO^+/-^ adult heterozygotes (*t*-test; mean ± SEM; ns, not significant; n=10).


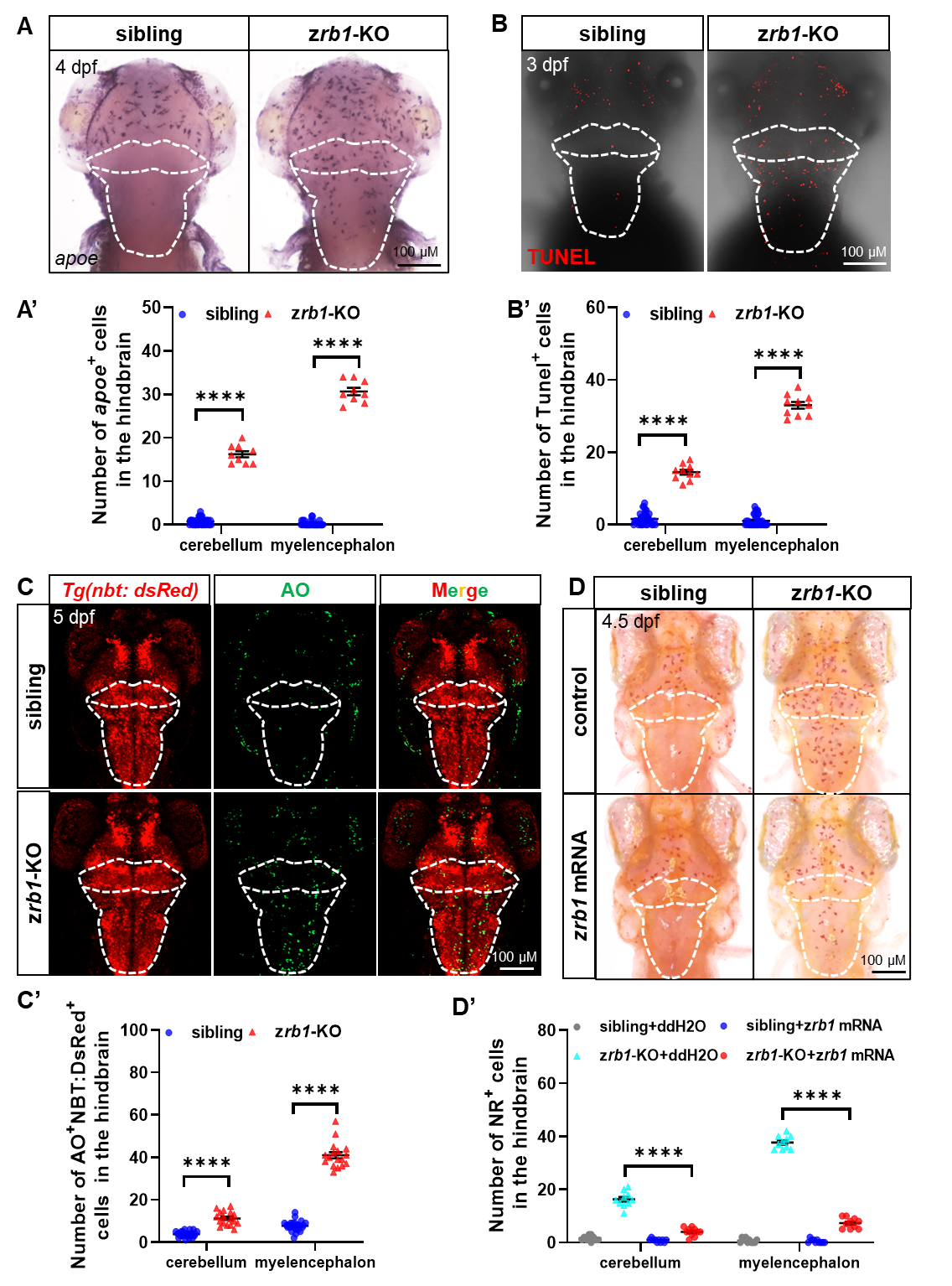


**Fig. S2.** **Neuronal apoptotic increased in z*rb1*-KO mutants. (A)** Whole-mount *in situ* hybridization of *apoe* (microglial marker) in the brain of siblings and z*rb1*-KO mutants at 4.5 dpf. **(A’)** The statistical plot of the number of *apoe*^+^ cells in the cerebellum and myelencephalon of siblings and z*rb1*-KO mutants (*t*-test; mean ± SEM; ^****^P<0.0001; n≥9). (**B)** TUNEL staining in siblings and z*rb1*-KO mutants at 5 dpf. The white dotted line outlines the cerebellum and myelencephalon. **(B’)** Quantification of TUNEL^+^ cells of the cerebellum and myelencephalon in siblings and z*rb1*-KO mutants of (B) (*t*-test; mean ± SEM; ^****^P<0.0001; n=10). **(C)** Co-staining of AO signals (green) and *Tg(nbt:dsRed)* of siblings and z*rb1*-KO mutants at 5 dpf. The white dotted line outlines the cerebellum and myelencephalon. The white arrows indicate the apoptotic cells. **(C’)** The statistical plot of the number of AO^+^/NBT-dsRed^+^ cells in the cerebellum and myelencephalon of siblings and z*rb1*-KO mutants (*t*-test; mean ± SEM; ^****^P<0.0001; n≥17). **(D)** NR staining in 3 dpf siblings and z*rb1*-KO mutants after injecting with ddH2O and z*rb1* mRNA. **(D’)** NR^+^ cells of the cerebellum and myelencephalon in all groups of (C) (one-way ANOVA; mean ± SEM; ^****^P<0.0001; n=10 each group).


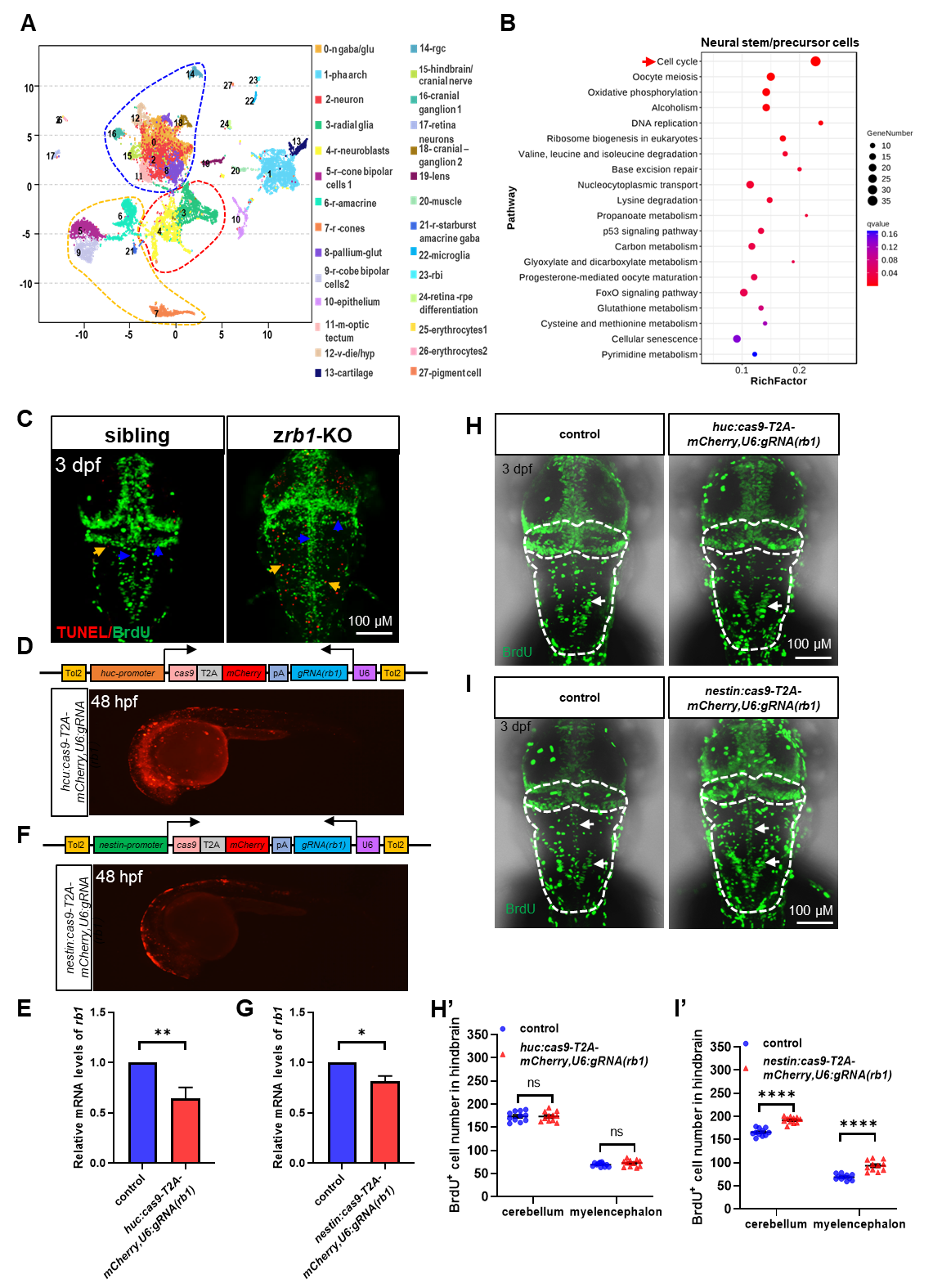


**Fig. S3. Rb1 regulates the proliferation and apoptosis of different neurons, respectively. (A)** An unsupervised UMAP plot subdivides cells into four major cell types and includes 27 clusters. NSPCs: 3-radial glia and 4-retina neuroblasts (r-neuroblasts); post-mitotic neurons: 0-neurons glutamatergic/gabaergic (n-gaba/glu), 2-neurons, 8-pallium-glutamatergic (pallium-glu), 11-midbrain optic tectum (midbrain op), 12-ventral diencephalon/hypothalamus(v-die/hyp), 14-retinal ganglion cell (rgc); 15-hindbrain/cranial nerve and 16/18-cranial ganglion; optic neurons: 5/9-retina cone bipolar cells (r-cone bipolar cells), 6-retina amacrine (r-amacrine), 7-retina-cones (r-cones) and 21-retina starburst amacrine gabaergic (r-starburst amacrine-gaba); non-neuronal cells: 1-pharyngeal arch (pha arch), 10-epithelium, 13-cartilage, 17-retina neuron (retina), 19-lens, 20-muscle, 22-microglia, 23-rostral blood island (rbi), 25/26-erythrocytes and 27-pigment cell. **(B)** The top 20 functionally enriched KEGG pathways were found in the analysis of DEGs in NSPCs. The red arrows indicate the cell cycle pathway. **(C)** Co-localization of proliferating and apoptotic cells by prolonged BrdU treatment (label the cells that had proliferated within 2+2 h) and TUNEL assay. Yellow arrows indicate the apoptotic cells and blue arrows indicate the proliferation cell. **(D and F)** Schematic illustration of the constructs of the *huc:cas9-T2A-mCherry, U6:gRNA(rb1)* plasmid (D) or *nestin:cas9-T2A-mCherry, U6:gRNA(rb1)* plasmid (F) and fluorescence expression after microinjection at 48 hpf. **(E and G)** Relative expression of *rb1* in whole brain from 3 dpf embryos after injecting with *huc:cas9-T2A-mCherry, U6:gRNA(rb1)* plasmid (E) or *nestin:cas9-T2A-mCherry, U6:gRNA(rb1)* plasmid (G) by qPCR analysis (*t*-test; mean ± SEM; **P<0.01; *P<0.05; n=30). **(H and I)** Dorsal views of BrdU staining after wild-type microinjection of *huc:cas9-T2A-mCherry, U6:gRNA(rb1)* (H) plasmid and *nestin:cas9-T2A-mCherry, U6:gRNA(rb1)* (I) plasmid*.* The white dotted line outlines the cerebellum and myelencephalon. The white arrows indicate the proliferating cells. **(H’ and I’)** The statistical analysis of BrdU^+^ cells in the cerebellum and myelencephalon between the control group and *huc:cas9-T2A-mCherry, U6:gRNA(rb1)* (H’) plasmid and *nestin:cas9-T2A-mCherry, U6:gRNA(rb1)* (I’) plasmid (*t*-test; mean ± SEM; ^****^P<0.0001; ns, not significant; n≥10).


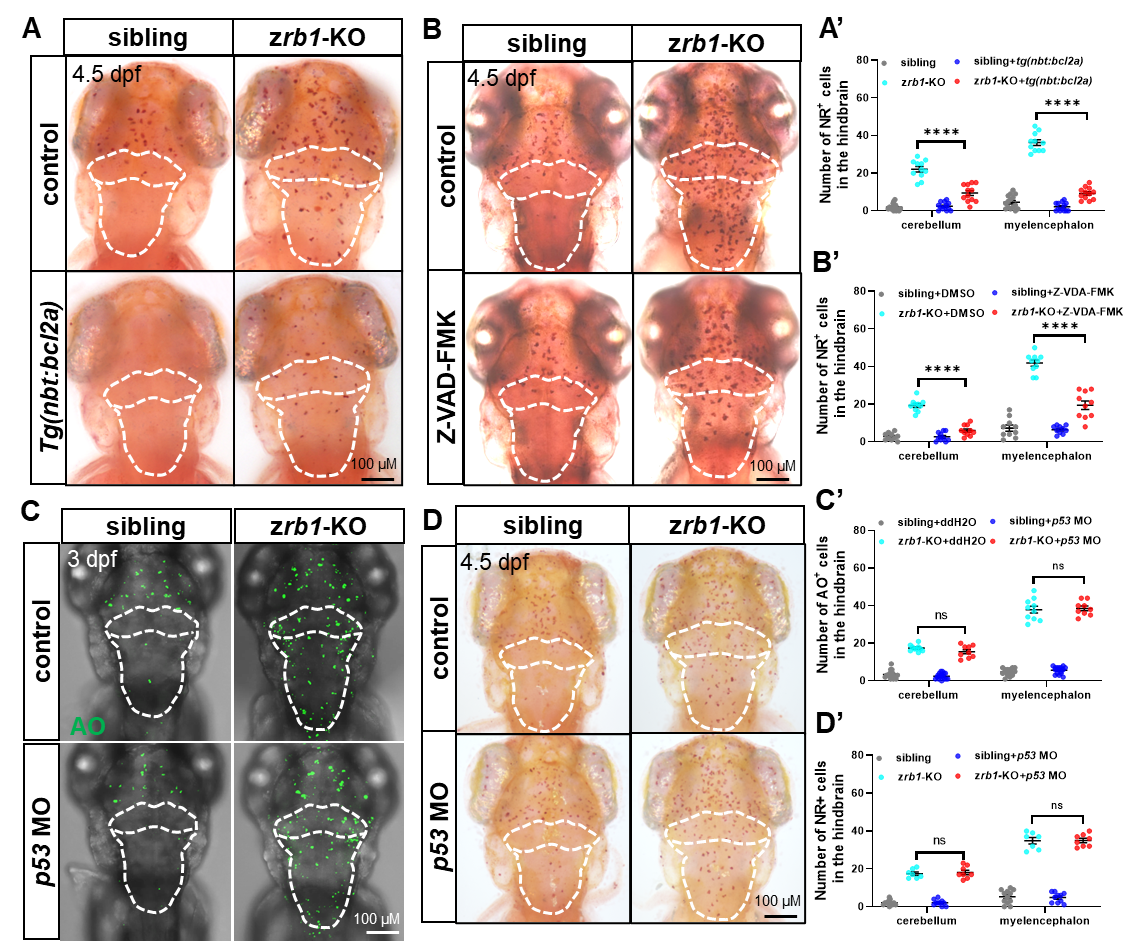


**Fig. S4.** **The Rb1 regulates post-mitotic neuron apoptosis through the *bcl2a/caspase* pathway. (A and B)** Stained by NR to visualize microglia in the cerebellum and myelencephalon at 4.5 dpf after overexpression of the *bcl2a* (A) and treated with pan-caspase inhibitor Z-VAD-FMK (B) of siblings and z*rb1*-KO mutants at 4.5 dpf. The white dotted line outlines the cerebellum and myelencephalon. **(A’ and B’)** The statistical plot of NR^+^ cells in the cerebellum and myelencephalon at 4.5 dpf after overexpression of the *bcl2a* (A’) and treated with pan-caspase inhibitor Z-VAD-FMK (B’) in siblings and z*rb1*-KO mutants (*t*-test; mean ± SEM; ****P<0.0001; n≥10). **(C and D)** AO staining (C) and NR staining (D) in siblings and z*rb1*-KO mutants after being injected with *p53* MO. The white dotted line outlines the cerebellum and myelencephalon. **(C’ and D’)** Quantification of AO^+^ cells (C’) and NR^+^ cells (D’) of the cerebellum and myelencephalon in siblings and z*rb1*-KO mutants after injected with *p53* MO (one-way ANOVA; mean ± SEM; ns, not significant; n≥7).


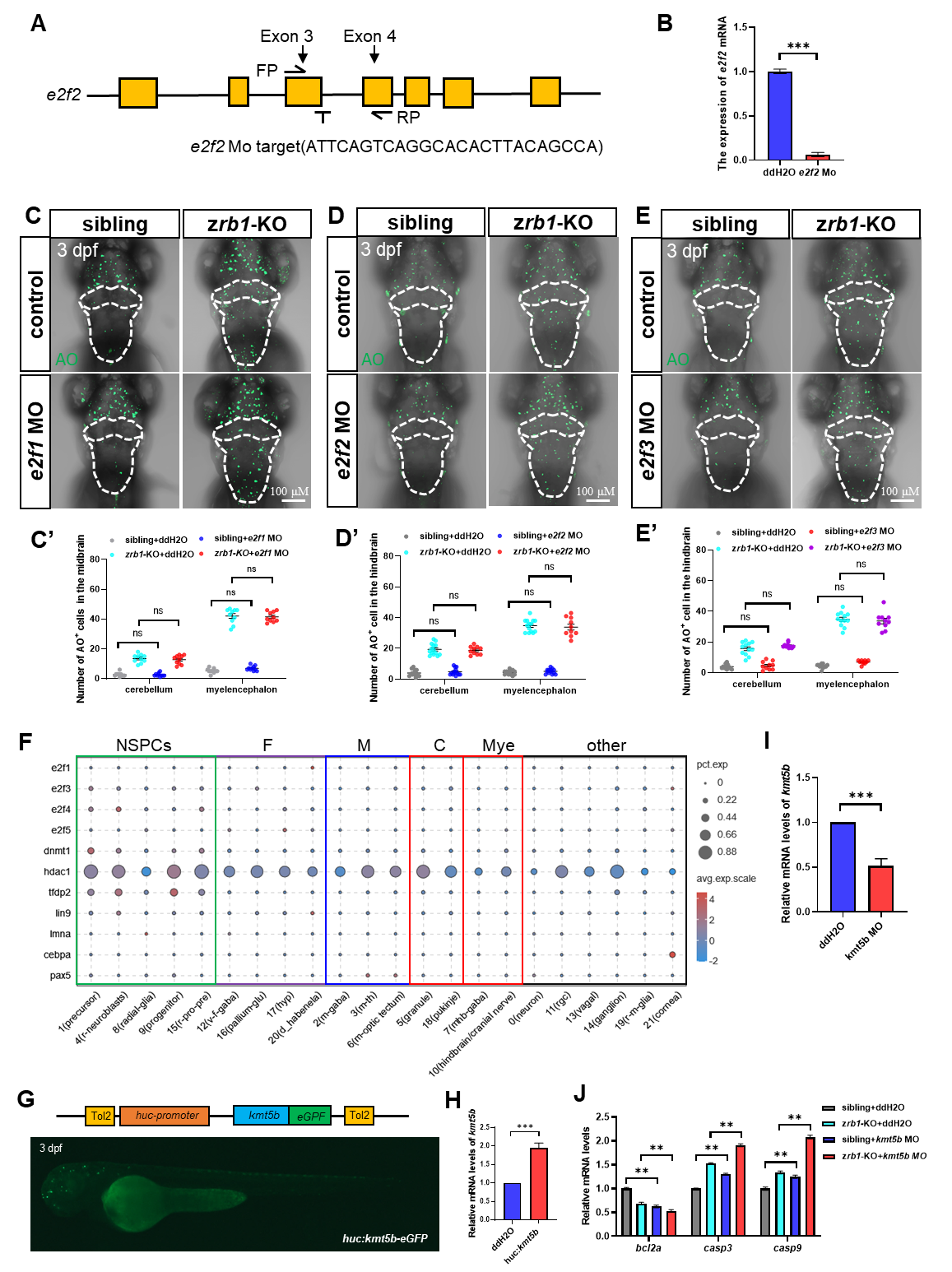


**Fig. S5. The Rb1 regulates post-mitotic neuron apoptosis through the Kmt5b-*bcl2a/caspase* pathway. (A)** The zebrafish *e2f2* gene structure. Exons are indicated by yellow boxes. Location and sequence of the splice morpholino target site for the *e2f2* gene. FP/RP, *e2f2* qPCR forward/reverse primers. **(B)** Relative expression of *e2f2* in controls and *e2f2* morphants at 3 dpf by qPCR analysis (*t-test*; mean ± SEM; ***P<0.001; n=30). **(C-E)** Co-staining of acridine orange (AO) signals (green) in 3 dpf sibling embryos and z*rb1*-KO mutants after injecting with control, and *e2f1* MO (C), *e2f2* MO (D), and *e2f3* MO (E). **(C’-E’)** Quantification of AO^+^cells of the hindbrain in control, and *e2f1* MO (C), *e2f2* MO (D), and *e2f3* MO (one-way ANOVA; mean ± SEM; ^***^P<0.001; n≥10 each group). **(F)** Dot plot showing the expression levels of *e2f1, e2f3, e2f4, e2f5, dnmt1, hdac1, tfdp2, lin9, lmna, cebpa, pax5* in six brain regions. The gray level represents the average expression; the dot size represents the percentage of cells expressing the marker genes. **(G)** Schematic illustration of the *huc:kmt5b-egfp* plasmid constructs and fluorescence expression after microinjection at 3 dpf. **(H and I)** Relative expression of *kmt5b* after injecting with *huc:kmt5b-egfp* plasmid and *kmt5b-*MO (*t*-test; mean ± SEM; ***P<0.001; n=30). **(J)** Relative expression of *bcl2a, casp3,* and *casp9* in siblings and z*rb1*-KO mutants from 3 dpf embryos by qPCR analysis (*t*-test; mean ± SEM; **P<0.01; n=30).
