# Supplementary material for: Role of RB1 in neurodegenerative diseases: inhibition of post-mitotic neuronal apoptosis via Kmt5b
Source: Cell Death Discov. 2024 Apr 18;10:182. doi: 10.1038/s41420-024-01955-y (PMC11026443; doi:10.1038/s41420-024-01955-y)
Supplement: Supplementary file 7 — original western blot [file 41420_2024_1955_MOESM7_ESM.docx]

Fig. 5 D original western blot

Anti-GPF


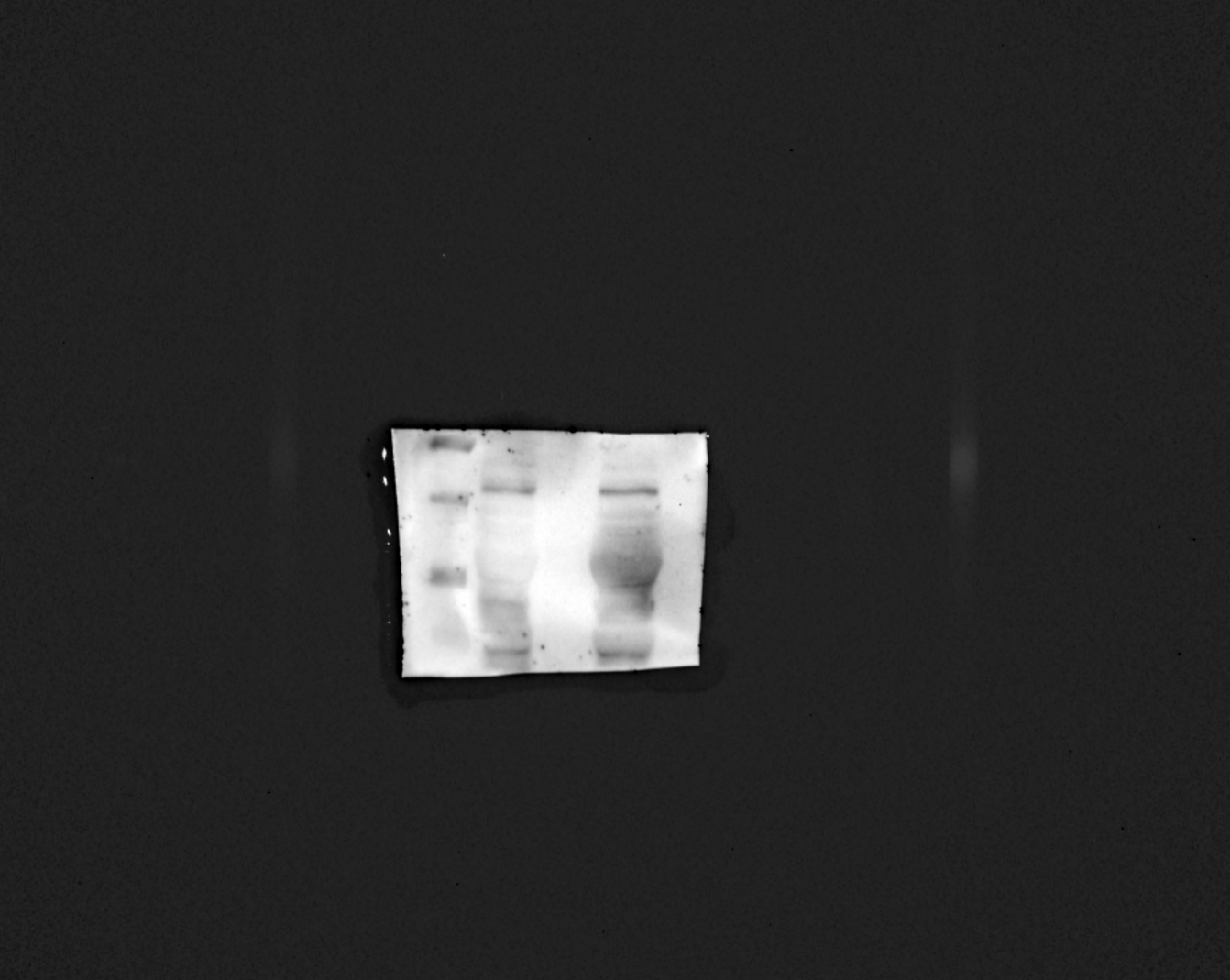


130kDa

Anti-mCherry


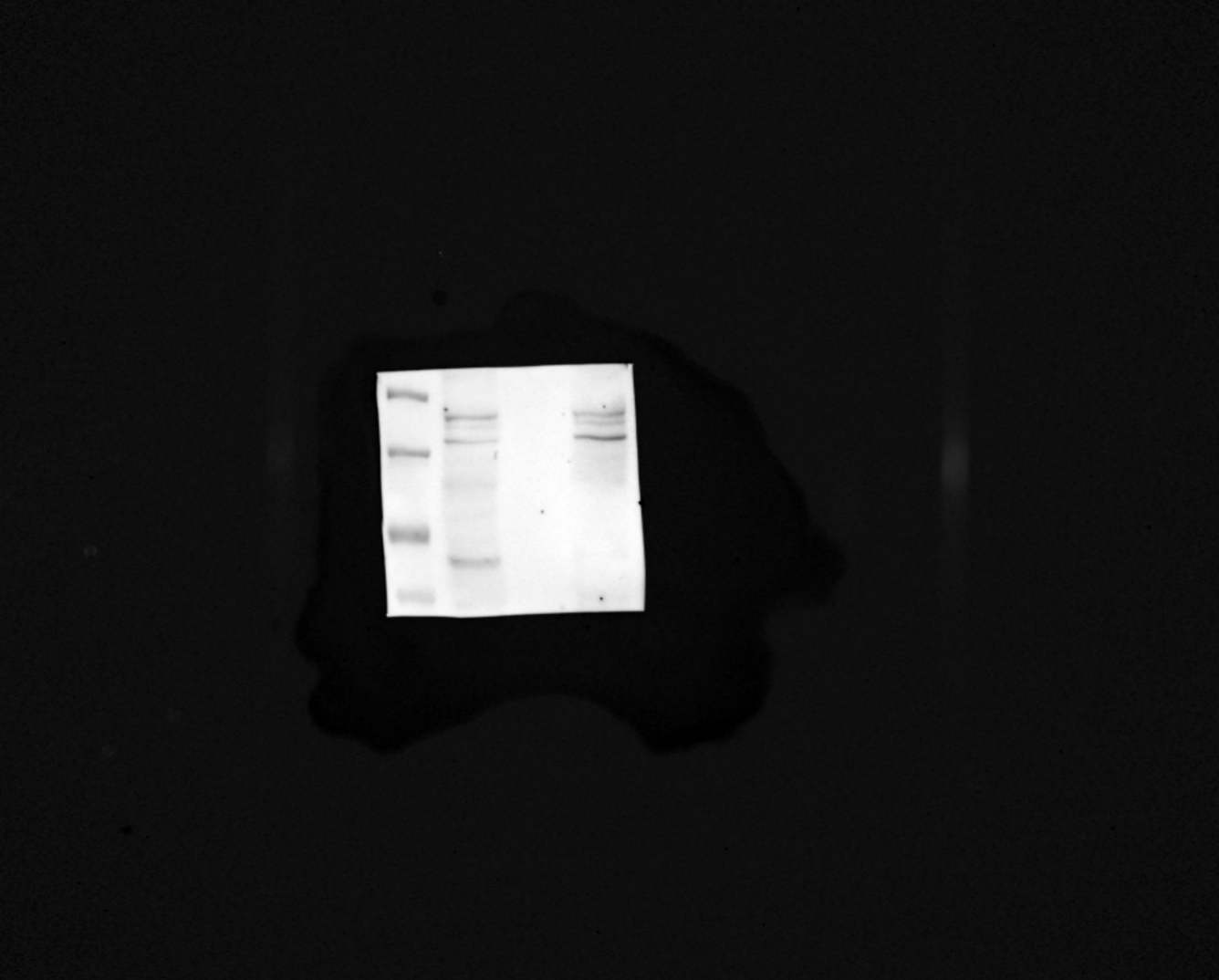


130kDa

Fig. 6 B original western blot

Anti-GPF


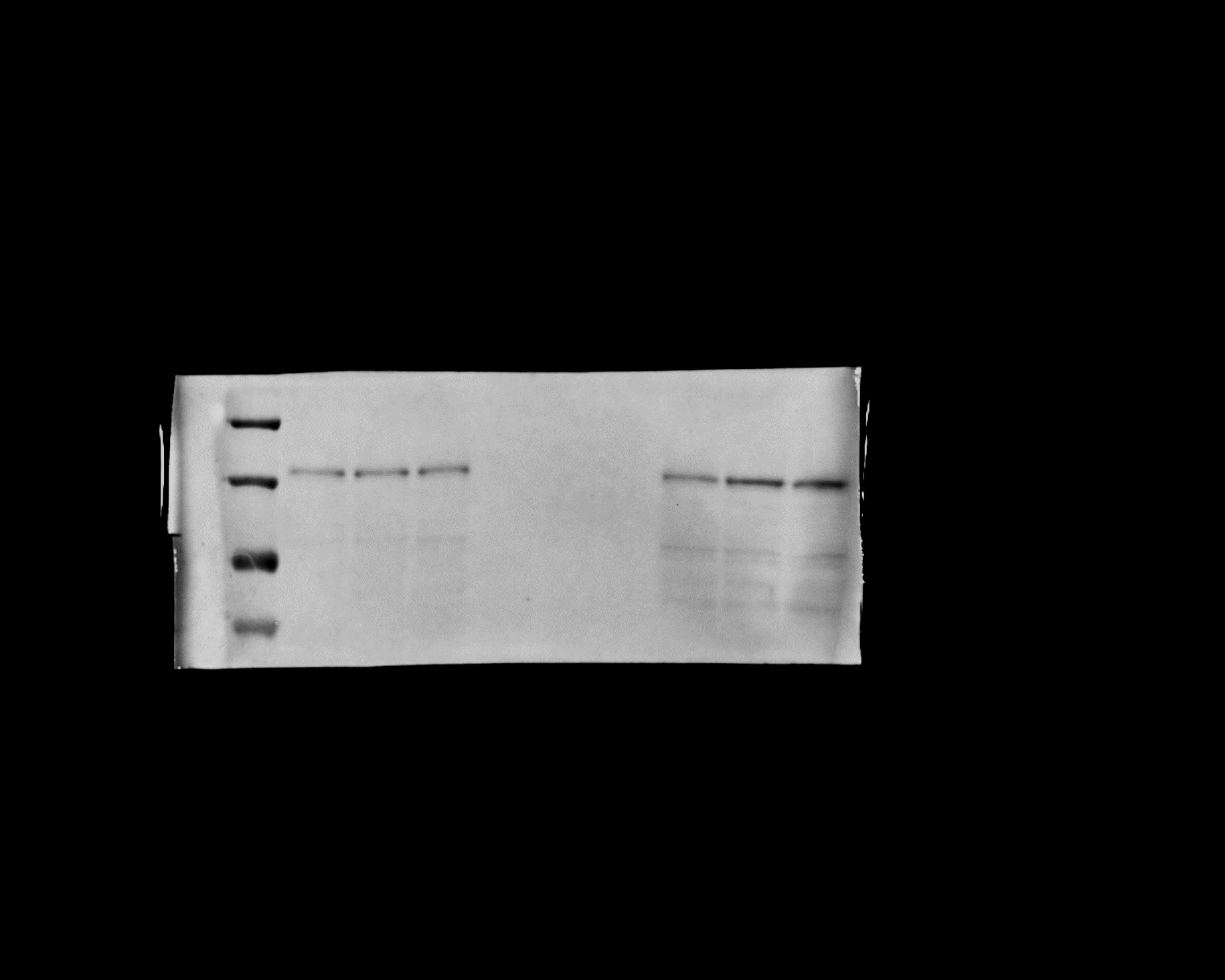


130kDa

Anti-mCherry


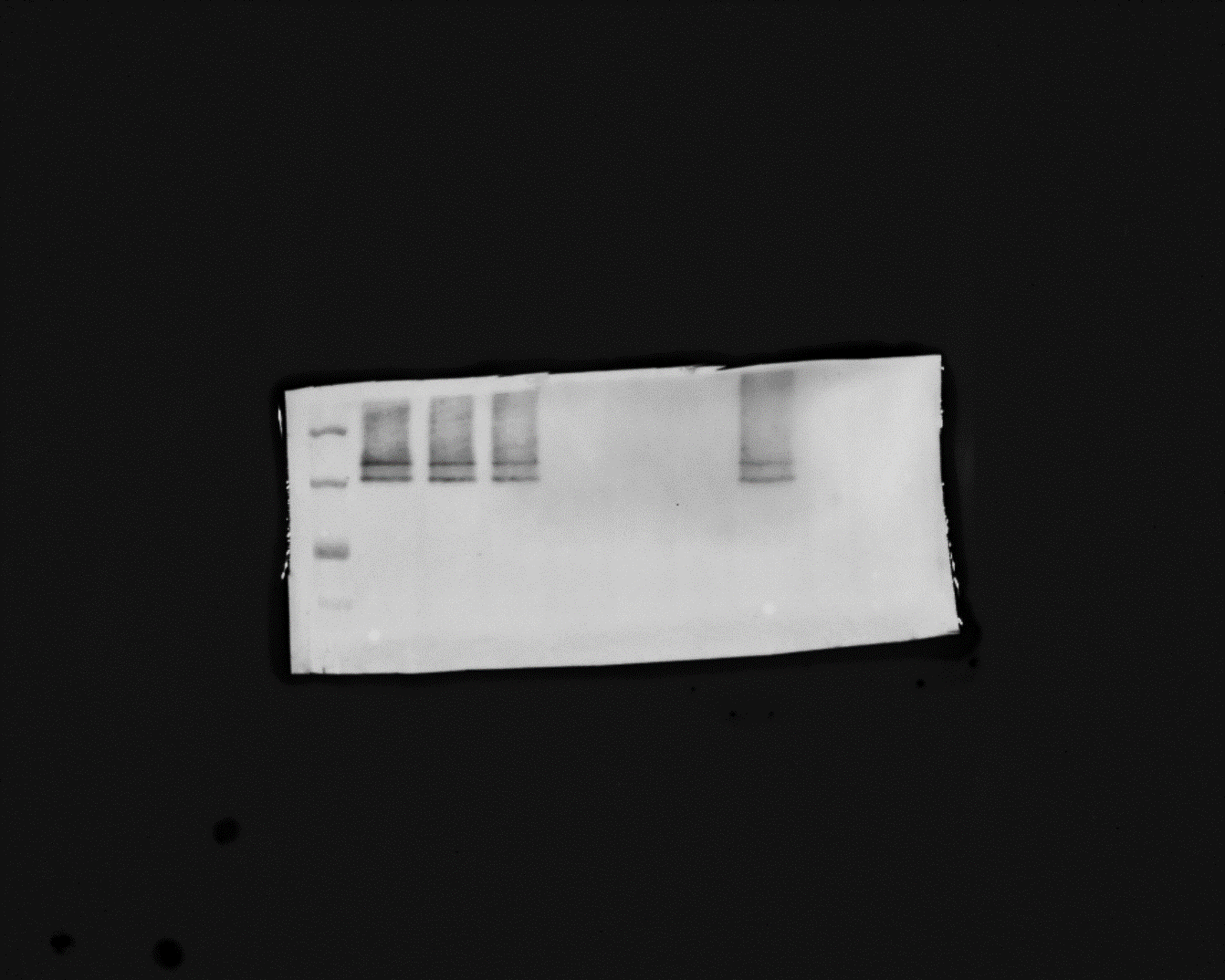


130kDa
